# Computational Study Protocol: Leveraging Synthetic Data to Validate a Benchmark Study for Differential Abundance Tests for 16S Microbiome Sequencing Data

**DOI:** 10.12688/f1000research.155230.1

**Published:** 2024-10-09

**Authors:** Eva Kohnert, Clemens Kreutz

**Affiliations:** 1 Institute of Medical Biometry and Statistics, Faculty of Medicine and Medical Center, University of Freiburg, Baden-Württemberg, Germany

**Keywords:** 16S, microbiome, differential abundance, simulation, synthetic data, benchmarking

## Abstract

**Background:**

The utility of synthetic data in benchmark studies depends on its ability to closely mimic real-world conditions and to reproduce results obtained from experimental data. Here, we evaluate the performance of differential abundance tests for 16S metagenomic data. Building on the benchmark study by Nearing et al. (1), who assessed 14 differential abundance tests using 38 experimental datasets in a case-control design, we validate their findings by generating synthetic datasets that mimics the experimental data. We will employ statistical tests to rigorously assess the similarity between synthetic and experimental data and to validate the conclusions on the performance of these tests drawn by Nearing et al. (1). This protocol adheres to the SPIRIT guidelines and is, to our knowledge, the first of its kind in computational benchmark studies.

**Methods:**

We replicate Nearing et al.’s (1) methodology, incorporating synthetic data simulated using two distinct tools, mirroring each of the 38 experimental datasets. Equivalence tests will be conducted on 43 data characteristics comparing synthetic and experimental data, complemented by principal component analysis for overall similarity assessment. The 14 differential abundance tests will be applied to both synthetic and experimental datasets, evaluating the consistency of significant feature identification and the number of significant features per tool. Correlation analysis and multiple regression will explore how differences between synthetic and experimental data characteristics may affect the results.

**Conclusions:**

Synthetic data enables the validation of findings through controlled experiments. We assess how well synthetic data replicates experimental data, validate previous findings and delineate the strengths and limitations of synthetic data in benchmark studies. Moreover, to our knowledge this is the first computational benchmark study to systematically incorporate synthetic data for validating differential abundance methods while strictly adhering to a pre-specified study protocol following SPIRIT guidelines, contributing significantly to transparency, reproducibility, and unbiased research.

## Protocol

**Table of Contents**

***Introduction***

**Background and rationale {6a}**

**Objectives {7}**

**Trial design {8}**

**Summary Table**

***Methods***

**Study population/participants**

**Study setting {9}**

**Eligibility criteria {10}**

**Who will take informed consent? {26a}**

**Additional consent provisions for collection and use of participant data and biological specimens {26b}**

***Interventions***

**Explanation for the choice of comparators {6b}**

**Intervention description {11a}**

**Criteria for discontinuing or modifying allocated interventions {11b}**

**Strategies to improve adherence to interventions {11c}**

**Relevant concomitant care permitted or prohibited during the trial {11d}**

**Provisions for post-trial care {30}**

***Outcomes {12}***

**Participant timeline {13}**

**Sample size {14}**

**Recruitment {15}**

**Assignment of interventions: allocation**

**Sequence generation {16a}**

**Concealment mechanism {16b}**

**Implementation {16c}**

**Assignment of interventions: Blinding**

**Who will be blinded {17a}**

**Procedure for unblinding if needed {17b}**

***Data collection and management***

**Plans for assessment and collection of outcomes {18a}**

**Plans to promote participant retention and complete follow-up {18b}**

**Data management {19}**

**Confidentiality {27}**

**Plans for collection, laboratory evaluation and storage of biological specimens for genetic or molecular analysis in this trial/future use {33}**

***Statistical methods {20}***

**Data monitoring committee {21a}**

**Statistical methods for primary and secondary outcomes {20a}**

**Interim analyses {21b}**

**Methods for additional analyses (e.g. subgroup analyses) {20b}**

**Methods in analysis to handle protocol non-adherence and any statistical methods to handle missing data {20c}**

**Plans to give access to the full protocol, participant level-data and statistical code {31c}**

***Timeline***

***Oversight and monitoring***

**Composition of the coordinating centre and trial steering committee {5d}**

**Composition of the data monitoring committee, its role and reporting structure {21a}**

**Adverse event reporting and harms {22}**

**Ancillary and post-trial care {30}**

**Frequency and plans for auditing trial conduct {23}**

**Plans for communicating important protocol amendments to relevant parties (e.g. trial participants, ethical committees) {25}**

***Dissemination policy {31a}***

***Discussion***

***Abbreviations***

***Declarations***

**Acknowledgements**

**Authors’ contributions {31b}**

**Availability of data and materials {29}**

**Ethics and consent {24}**

**Consent for publication {32}**

**Authors’ information (optional)**

***Data availability***

***References***

***Study status***

Note: To achieve a rigorous methodology, this protocol adheres to an established standard and checklist for Standard Protocol Items: Recommendations for Interventional Trials (SPIRIT). ^2^ The numbers in curly brackets in this protocol refer to SPIRIT checklist item numbers. The order of the items has been modified to group similar items. Since using a standardized terminology for study designs is essential, we also formulate our protocol using standard terminology, i.e. terms such as study population, comparator, intervention, outcome, modification, inclusion and exclusion.

**Table 1.** Administrative information summary.

Title {1}	Computational Study Protocol: Leveraging Synthetic Data to Validate a Benchmark Study for Differential Abundance Tests for 16S Microbiome Sequencing Data.
Trial registration {2a and 2b}.	Currently, there exists no registry tailored specifically to computational benchmark studies. This study does not involve interventions on humans or animals; rather, it exclusively incorporates publicly accessible sequencing data.
Protocol version {3}	August 09, 2024, Version 2
Grant information (Funding {4})	The author(s) declared that no third-party grants were involved in supporting this work.
Author details {5a}	Eva Kohnert: Institute of Medical Biometry and Statistics, Faculty of Medicine and Medical Center – University of Freiburg, Germany Clemens Kreutz: Institute of Medical Biometry and Statistics, Faculty of Medicine and Medical Center – University of Freiburg, Germany.
Name and contact information for the trial sponsor {5b}	n/a: There is no sponsor.
Role of sponsor {5c}	n/a: There is no sponsor.

## Introduction

### Background and rationale {6a}

Differential abundance (DA) analysis of metagenomic microbiome data has emerged as a pivotal tool in understanding the complex dynamics of microbial communities across various environments and host organisms. ^3– 5^ Microbiome studies are crucial for identifying specific microorganisms that differ significantly in abundance between different conditions, such as health and disease states, different environmental conditions, or before and after a treatment. The insights we gain from analyzing the differential abundance of microorganisms are critical to understanding the role that microbial communities play in environmental adaptations, disease development and health of the host. ^6^ Refining statistical methods for the identification of changes in microbial abundance is essential for understanding how these communities influence disease progression and other interactions with the host, which then enables new strategies for therapeutic interventions and diagnostic analyses. ^7^

The statistical interpretation of microbiome data is notably challenged by its inherent sparsity and compositional nature. Sparsity refers to the disproportionate proportion of zeros in metagenomic sequencing data and requires tailored statistical methods, ^8, 9^ e.g. to account for so-called structural zeros that originate from technical limitations rather than from real absence. ^10^ Additionally, due to the compositional aspect of microbiome data regulation of highly abundant microbes can lead to biased quantification of low-abundant organisms. ^11^ Such bias might be erroneously interpreted as apparent regulation that is mainly due to the compositional character of the data. Such characteristics of microbiome data have a notable impact on the performance of common statistical approaches for DA analysis, delimits their applicability for microbiome data and poses challenges about the optimal selection of DA tests.

A number of benchmark studies have been conducted to evaluate the performance of DA tests in the analysis of microbiome data. ^12– 15^ However, the results show a very heterogeneous picture and clear guidelines or rules for the appropriate selection of DA tests have yet to be established. In order to assess and contextualize the findings of those studies, additional benchmarking efforts using a rigorous methodology, ^16, 17^ as well as further experimental and synthetic benchmark data sets are essential.

Synthetic data is frequently utilized to evaluate the performance of computational methods because for such simulated data the ‘correct’ or ‘true’ answer is known and can be used to assess whether a specific method can recover this known truth. ^16^ Moreover, characteristics of the data can be changed to explore the relationship between data characteristics such as effect size, variability or sample size and the performance of the considered methods. Several simulation tools have been introduced for generating synthetic microbiome data. ^18– 23^ They cover a broad range of functionality. For example, MB-GAN ^22^ leverages generative adversarial networks to capture complex patterns and interactions present in the data, while metaSPARSim, ^18^ sparseDOSSA2 ^19^ or nuMetaSim ^24^ employ different statistical models to generate microbiome data. Introducing a new simulation tool typically involves demonstrating its capacity to replicate key data characteristics. Nonetheless, an ongoing question persists regarding the feasibility of validating findings derived from experimental data when synthetic data, generated to embody the characteristics of the experimental data, is used in its place.

Here we refer to the recent high-impact benchmark study of Nearing et al. ^1^ in which the performance of a comprehensive set of 14 DA tests applied to 38 experimental 16S microbiome data sets was compared. This 16S microbiome sequencing data is used to study communities in various environments, here from human gut, soil, wastewater, freshwater, plastisphere, marine and built environments. The data sets are presented in a two group design for which DA tools are applied to identify variations in species abundances between the groups.

In this validation study we replicate the primary analysis conducted in the reference study by substituting the actual datasets with corresponding synthetic counterparts. The objective is to explore the validity of the main findings from the reference benchmark study when the analysis workflow is repeated with an independent implementation and with synthetic data, generated to recapitulate the characteristics of the original real data.

### Objectives {7}

Aim 1: Synthetic data, simulated based on an experimental template, overall reflect main data characteristics.

Aim 2: Study results from Nearing et al. can be validated using synthetic data, simulated based on corresponding experimental data.

### Trial design {8}

Aim 1: Exploratory comparative study

Aim 2: Confirmatory benchmark study

### Summary Table

**Table 2.** Summary of hypotheses and the stasticial analyses used for evaluation.

Research question	Hypothesis	Statistical analyses	Confirmation criteria
Aim 1: Can state of the art simulation tools for metagenomics data realistically generate synthetic data across a broad range of simulation templates?	Main data characteristics calculated from synthetic data are equivalent to experimental templates.	Equivalence tests, i.e. two one-sided one-sample t-tests for each data characteristic as implemented in the TOSTER R-package. PCA of all data characteristics and equivalence test for Euclidean distances in 2D.	We interpret a p-value < 0.05 for rejecting the null hypothesis “non-equivalence” as significant and then conclude that the respective data characteristic is equivalent.
Aim 2: Can conclusions based on performance outcomes (proportion of significant taxa and overlap across DA tests) from 16S microbiome sequencing data be validated with synthetic data, simulated after calibration based on the used experimental data?	Hypotheses 1: 13 extracted outcomes from (1) concerning the overlap of significant features across exp. data sets and DA test can be confirmed based on their corresponding simulations. Hypothesis 2: 14 extracted outcomes from (1) concerning the proportion of significant features identified across multiple DA tools can be confirmed based on their corresponding simulations.	For 23/27 hypotheses: Estimating the proportion P where the hypotheses are fulfilled by counting, and calculation of 95% confidence intervals a) for independent observations based on the SE formula b) for dependent observation using bootstrap. For 2/27 hypotheses: 2-way ANOVA For 1/27 hypothesis: mean of Kolmogorov-Smirnov test statistic For 1/27 hypothesis: visualization by histograms.	For each hypothesis, we specified individual confirmation thresholds. In 18/27 cases, we use a 95% threshold as criterion for the estimated proportion of cases, where the hypothesis is valid. We check these criteria to be fulfilled by considering the 95% CI. For ANOVA and Kolmogorov-Smirnov tests, we also specify individual confirmation criteria.

## Methods

### Study population/participants

In the context of our benchmark study, the study population is given by the experimental data sets from the reference study. ^1^

### Study setting {9}

Where possible, the study is conducted analogously to the benchmark study conducted by Nearing et al., ^1^ e.g. the same data and primary outcomes will be used.

All data sets as provided by Nearing et al. ^1^ will be included in the study. We employ two published simulation tools, metaSPARSim ^18^ and sparseDOSSA2, ^19^ which have been developed for simulating microbial abundance profiles as they are generated by 16s sequencing.

We also apply the same DA tests as in ^1^ and implementation in the R statistical programming language. In order to provide the most valuable results for the bioinformatics community, the latest versions of these implementations will be used.

### Eligibility criteria {10}

**Inclusion criteria**

We will include the same experimental data sets and DA tests as in Ref. 1.

**Exclusion criteria**

There are no exclusion criteria for the data sets.

### Who will take informed consent? {26a}

n/a: Data is publicly available, there is no need to obtain consent.

### Additional consent provisions for collection and use of participant data and biological specimens {26b}

n/a: Data is publicly available, there is no need to obtain consent.

## Interventions

### Explanation for the choice of comparators {6b}

For aim 1, the comparator are 43 data characteristics calculated from the 38 experimental data sets. These characteristics are chosen such that they provide a comprehensive description of count matrices and enabling unbiased comparison between experimental and synthetic data sets. They cover for example information about the sparsity in a data set, mean-variance trends of features (taxa), or effect sizes between groups of samples. Tables 4 and 5 provide a detailed summary of all data characteristic and how they are calculated.

**Table 3.** Study status.

Version	Date	Changes made	Reason for changes
1	February 13, 2024	Initial submission as registered report to PLOS Biology and PLOS ONE (not accepted)	Initial version Link to initial version: https://nxc-fredato.imbi.uni-freiburg.de/s/o6TsmZBMdngtamp
2	August 09, 2024	Clarify data used for the hypotheses; some minor text changes (no methodological changes)	Hypotheses align to conclusions in Nearing et al.; make some sentences more precise Link to version with edits: https://nxc-fredato.imbi.uni-freiburg.de/s/jSRNoQxYzk5E6LW
3	August 13, 2024	Change order and naming of sections	Naming of protocoll sections need to align with F1000 requirements Link to version with edits: https://nxc-fredato.imbi.uni-freiburg.de/s/j5ibzbXMwW3Ssj9

Summary of all protocol versions and the corresponding changes.

**Table 4.** Calculation of data characteristics in R.

Name of data characteristic	Name in matrix (data.prop) summarizing all data characteristics	Calculation in R	Dimension
dat.cpm Counts per million normalized and log transformed data		edgeR::cpm (dat, log=TRUE, prior.count = 1)	mxn
Feature sparsity	data.prop$P0_feature	apply (dat==0,1,sum)/ncol (dat)	m
Sample sparsity	data.prop$P0_sample	apply (dat==0,2,sum)/nrow (dat)	n
Feature mean abundance	data.prop$mean_log2cpm	apply (dat.cpm, 1,mean,na.rm=T)	m
Feature median abundance	data.prop$median_log2cpm	apply (dat.cpm,1, median,na.rm=T)	m
Feature variance	data.prop$var_log2cpm	apply (dat.cpm, 1, var)	m
Library size	data.prop$lib_size	colSums (dat)	n
Sample means	data.prop$sample_means	apply (dat,2,mean)	n
Sample correlation	data.prop$corr_sample	cor (dat, dat, method="spearman",use="na.or.complete")	nxn
Feature correlation	data.prop$corr_feature	cor(t (dat), t (dat), method="spearman",use="na.or.complete")	mxm

Data characteristics that are vectors or matrices. These are used in Table 3 to calculate the final 43 integer value characteristics. Here, dat is a (mxn)-Matrix, with features as rows and samples as columns.

**Table 5.** Final integer values data characteristic and their calculation in R.

Name of data characteristic	Calculation in R
Number of features	nrow (dat)
Number of samples	ncol (dat)
Sparsity of data set	sum (dat==0)/length (dat)
Median of data set	median (dat,na.rm=TRUE)
95th Quantile	quantile (dat,probs=.95)
99th Quantile	quantile (dat,probs=.99)
Mean library size	mean (colSums (dat),na.rm = T)
Median library size	median (colSums (dat),na.rm = T)
Standard deviation library size	sd (colSums (dat),na.rm = T)
Coefficient of variation of library size	sd (colSums (dat),na.rm = T)/mean (colSums (dat),na.rm = T)*100
Maximum library size	max (colSums (dat),na.rm = T)
Minimum library size	min (colSums (dat),na.rm = T)
Read depth range between samples	diff (range (colSums (dat),na.rm = T))
Mean sample richness	mean (colSums (dat>0), na.rm=T)
Spearman correlation library size with P0*(sample)	cor (data.prop$P0_sample, data.prop$lib_size, method=“spearman”)
Bimodality of feature correlations	bimodalIndex (matrix (data.prop$corr_feature,nrow=1))$BI
Bimodality of sample correlations	bimodalIndex (matrix (data.prop$corr_sample,nrow=1))$BI
Mean of all feature means	mean (data.prop$mean_log2cpm,na.rm=T)
SD of all feature means	sd (data.prop$mean_log2cpm,na.rm=T)
Median of all feature means	median (data.prop$median_log2cpm,na.rm=T)
SD of all feature medians	sd (data.prop$median_log2cpm,na.rm=T)
Mean of all feature variances	mean (data.prop$var_log2cpm,na.rm=T)
SD of all feature variances	sd (data.prop$var_log2cpm,na.rm=T)
Mean of all sample means	mean (data.prop$sample_means,na.rm=T)
SD of all sample means	sd (data.prop$sample_means,na.rm=T)
Mean of sample correlation matrix	mean (data.prop$corr_sample,na.rm=T)
SD of sample correlation matrix	sd (data.prop$corr_sample,na.rm=T)
Mean of feature correlation matrix	mean (data.prop$corr_feature,na.rm=T)
SD of feature correlation matrix	sd (data.prop$corr_feature,na.rm=T)
Mean-Variance relation: Linear component	res <-lm(y~x+I(x ^2^),data=data.frame(y=data.prop$var_log2cpm,x=data.prop$mean_log2cpm)) res$coefficients[2]
Mean-Variance relation: Quadratic component	res=lm(y~x+I(x ^2^),data=data.frame(y=data.prop$var_log2cpm,x=data.prop$mean_log2cpm)) res$coefficients[3]
Slope feature sparsity vs. feature mean	res=lm(y~slope,data=data.frame (slope=data.prop$P0_feature-1,y=data.prop$mean_log2cpm)) res$coefficients[2]
Clustering of features	coef.hclust (hcluster (dat.tmp))
Clustering of samples	coef.hclust (hcluster(t (dat.tmp)))
Sample sparsity	apply (dat==0,2,sum)/nrow (dat)
Library sizes	colSums (dat)
Mean read depths	apply (dat,2,mean)
Feature sparsity	apply (dat==0,1,sum)/ncol (dat)
Feature mean intensity	apply (dat.cpm, 1,mean)
Feature median intensity	apply (dat.cpm,1, median)
Feature variances	apply (dat.cpm, 1, var)
Sample correlations	cor (dat, dat, method="spearman")
Feature correlations	calc_feature_corr(dat)

List of the 43 integer value characteristics and their calculation in R. P0 refers to ‘Percent of zeros’.

For aim 2, 14 differential abundance (DA) tests are applied to the experimental data (ALDEx2, ANCOM-II, corncob, DESeq2, edgeR, LEfSe, limma voom (TMM), limma voom (TMMwsp), MaAsLin2, MaSsLin2 (rare), metagenomeSeq, t-test (rare), Wilcoxon test (CLR), Wilcoxon test (rare)), i.e. the outcomes (number of significant features) calculated from the experimental data sets will serve as comparator. As in Ref. 1, we analyzed unfiltered data as well as data filtered with respect to features with a sufficient number of non-zero counts.

### Intervention description {11a}

The intervention consists of using synthetic data instead of experimental data.

For each of the 38 experimental data sets, synthetic data will be simulated. For the simulation two simulation tools (metaSPARSim ^18^ and sparseDOSSA2 ^19^) will be used. Simulation parameters are calibrated using the experimental data, such that the simulated data reflect the experimental data template. Both simulation approaches offer such a calibration functionality. Multiple (N=10) data realizations will be generated for each experimental data template to assess the impact of different realizations of simulation noise and to test for significant differences between interventions and the comparator.

For aim 1, the data characteristics will be computed for each of the synthetic and experimental data sets. For aim 2, 14 DA tests will be applied to the synthetic data generated in aim1.

### Criteria for discontinuing or modifying allocated interventions {11b}

**Figure 1.**
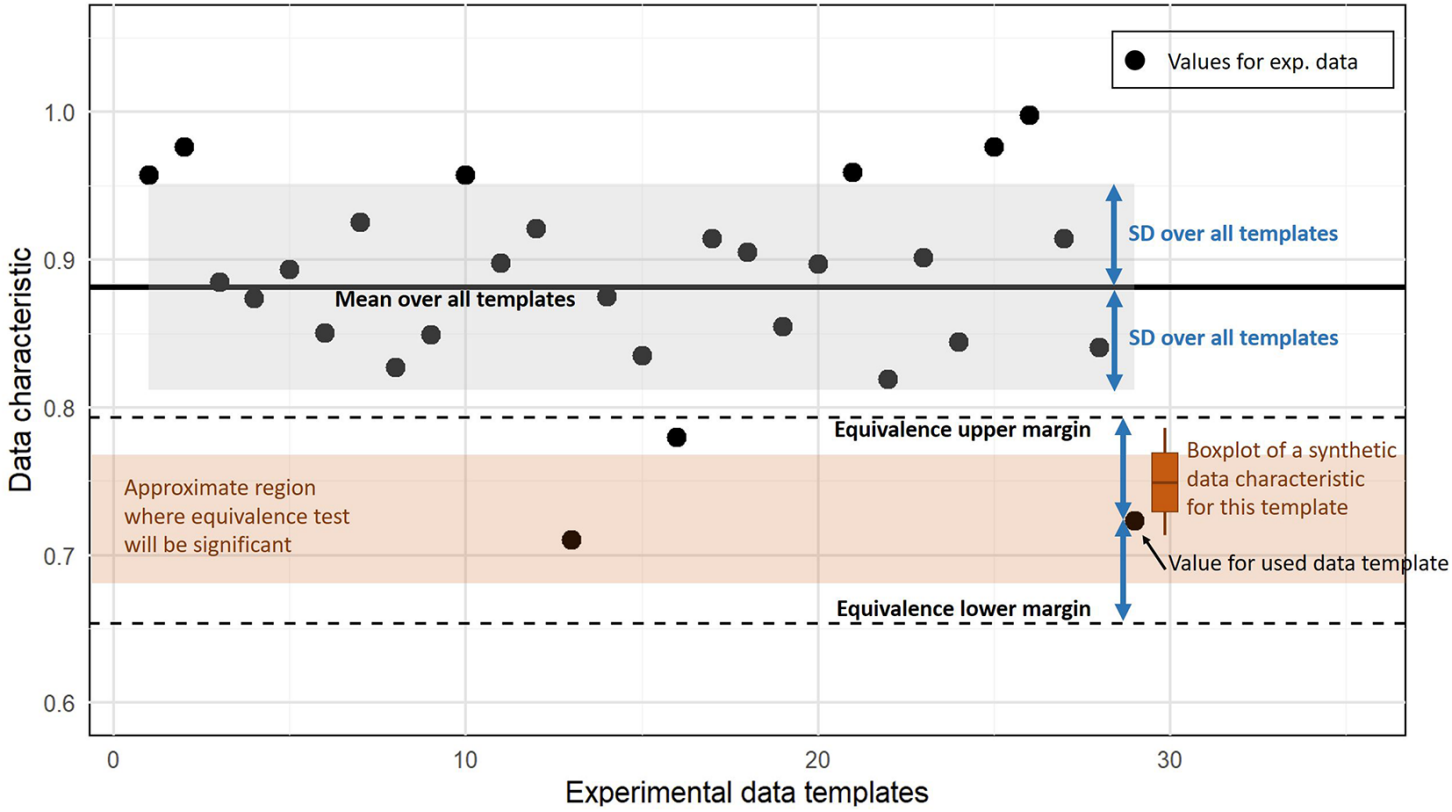
Illustration of assessing similarity based on an equivalence test. The black dots indicate a data characteristic computed for experimental data sets (here the proportion of zeros). Equivalence tests requires an interval that is considered as equivalent given by lower and upper margin bounds (dashed lines). We use the SD over all values from experimental templates to define these margins. The values computed from the synthetic data for a template are considered as equivalent if values below the lower margin and above the upper margin can be rejected according to the prespecified significance level. Depending on the variation of the characteristic for the synthetic data (here indicated by the boxplot), the average characteristic has to be inside a region (brown region) that is smaller than the interval between both margins.

For assessing the similarity of the synthetic data templates, we apply equivalence tests based on two one-sided t-tests as implemented in the TOSTER R-package with a 5% significance level. We use the SD of the respective values from all experimental data templates as lower and upper margins. Figure 1 illustrates the equivalence testing procedure for the proportion of zeros in the whole data set as an exemplary data characteristic. For equivalence testing, the combined null hypothesis that the tested values are below the lower margin or above the upper margin has to be rejected to conclude equivalence. This only occurs when the average data characteristic of synthetic data is inside both margins and not too close to those two bounds, i.e. the whole 95% CI interval of the estimated mean has to be between both margins.

**Modification by adjusting the proportions of zeros and effect sizes**

If equivalence tests fail, i.e. the synthetic data turns out to be partly unrealistic, we try to reduce the number of failed tests by adjusting two important characteristics, the proportion of zeros in the synthetic data sets, and the effect size, i.e., magnitude of differences between the two groups of samples.

Modifying the proportion of zeros will be performed by the following procedure for all synthetic data sets:

1. If the number of rows or columns of the experimental data template does not coincide, randomly add or delete columns and rows in the template.
2. Count the number of zeros that have to be added (or removed) for a simulated data set to obtain the same number as in the template.
3. If the simulation method does not generate data with matching order of features (i.e. rows), sort all rows of both count matrices according to row means.
4. Copy and replace an appropriate number of zeros (or non-zeros) one-by-one (i.e. with the same row and column indices) from the template to the synthetic data by randomly drawing those positions.
5. Reorder the rows to get the original ordering.
6. Check, whether the total number of failed equivalence test across all data templates is reduced.

Since we calibrate the simulation tools for both groups separately, all simulation parameters controlling the count distribution will be different in both groups. Therefore, we anticipate that the differences between both groups might be overestimated. We therefore try to make the simulation more realistic by modifying the effect size by the following procedure for all synthetic data sets:

1. Estimate the proportion of unregulated features from the results of all DA methods applied to the experimental data templates. This is done by the pi0est function in the qvalue R-package.
2. Calibrate the simulation tool by using all samples from both groups (then there is no difference between both groups of samples) and generate a synthetic data set without considering the assignment of samples to groups.
3. Replace an appropriate number of rows in the original synthetic data set by rows from the group-independent simulation.

In addition to both individual modifications, we also apply both modification. For the following analyses, we then use the synthetic data where most data characteristics are equivalent.

**Exclusion criteria**

**Figure 2.**
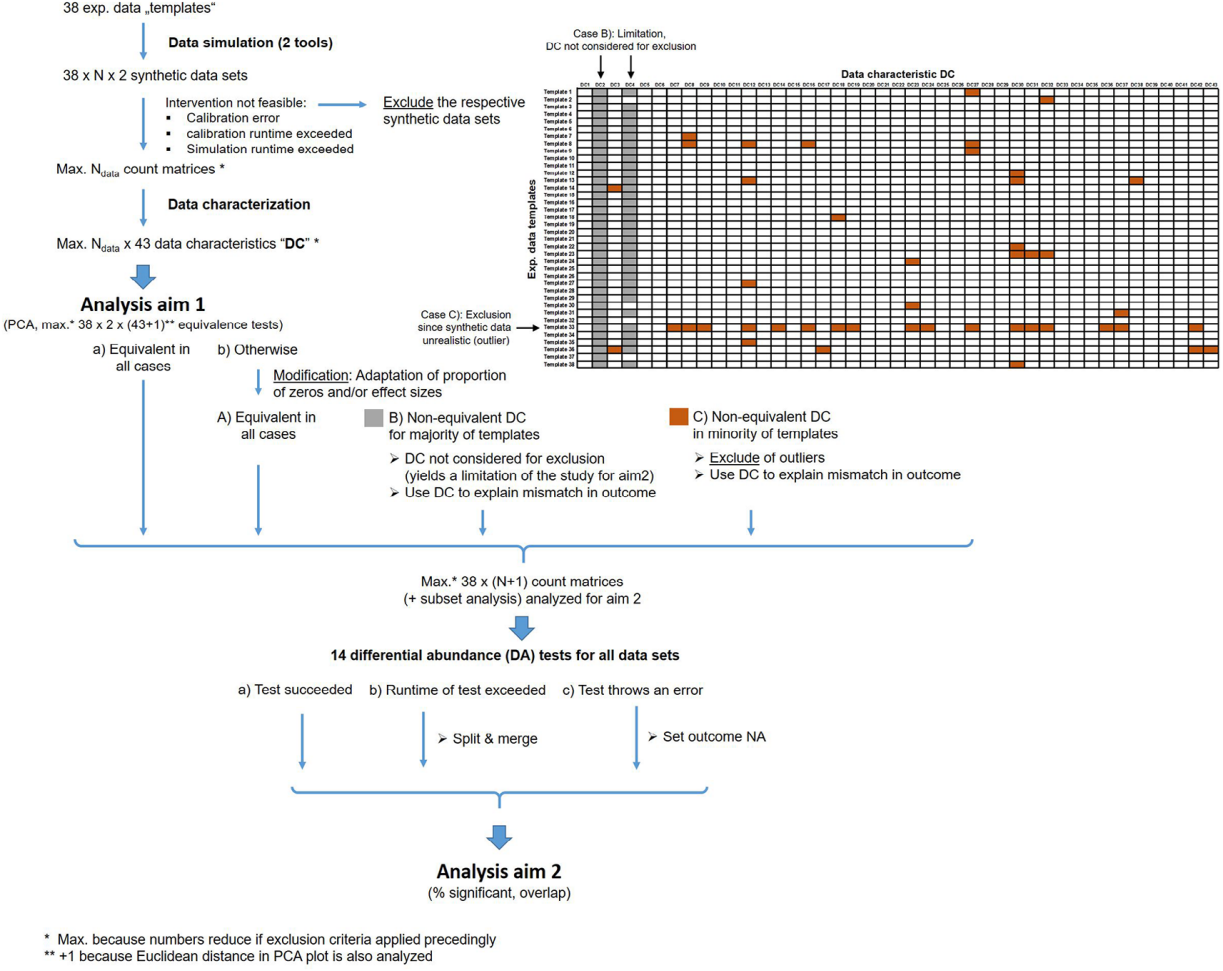
Overview about the analysis workflow and the exclusion/modification strategy. These criteria are applied to handle runtime issues, computation errors and unrealistic synthetic data.

In our study, we use experimental data as templates for generating synthetic data which are then analyzed by DA methods. At both levels, generation of synthetic data and applying DA methods, we define exclusion or modification criteria in order to handle exceeding runtimes, computation errors, and unrealistic data simulation. Figure 2 shows an overview about these exclusion and modification steps.

**Exclusion of simulation for a specific data template based on simulation performance**

A simulation tool will be excluded for a specific data template, if calibration of the simulation parameters is not feasible. We define feasibility by the following criteria:

1) Calibration succeeds without error message
2) The runtime of the calibration procedure is below 7 days (168 hours) for one data template
3) The runtime of simulating a synthetic data set is below 1 hour for one synthetic data set

All computations in this study will be performed on a Linux Debian x86_64 compute server with 64 AMD EPYC 7452 32-Core Processor CPUs. Although, we will run parts of the analyses in parallel mode, the specified computation times refer to runtimes on a single core.

**Exclusion of simulations for a specific data template based on deviating data properties**

For aim 2, we exclude synthetic data sets that are not similar enough to the experimental data sets used as templates. The goal of the following exclusion criterion is to exclude synthetic data sets that are overall strongly dissimilar from the experimental data template, without being too stringent since the simulation tools cannot perfectly resemble all data characteristics and therefore a slight or medium amount of dissimilarity has to be accepted. In general, dissimilarities are exploited to study the impact of those characteristics by investigating the association of such deviations with dissimilarity in outcomes. For assessing similarity, the data characteristics described before and specified in detail in Table 5 are used.

We expect that a few data characteristics are very sensitive in discriminating experimental and synthetic data. To prevent loss of too many data sets, such characteristics (highlighted in gray in Figure 2) are only considered for the investigation of association between mismatch in outcome and mismatch in data characteristics but not for exclusion.

Unrealistic synthetic data will be excluded for the primary analyses of the study using the remaining data characteristics. We define the exclusion criteria due to dissimilarity from its template by one of the following criteria:

1) The equivalence test based on Euclidean distance in the 2-dimensional PCA plot failed to indicate equivalence with the respective data templates. For equivalence testing, we use +/- 1 SD of the Euclidean distance over all exp. data templates as upper and lower margins.
2) We apply equivalence tests for all 43 data characteristics individually. We then only consider data characteristic which are not highly-discriminative. When counting non-equivalence of the remaining characteristics (highlighted in brown color in Figure 2) for a template, the synthetic data of those templates that appear as an outlier will be removed (see example in Figure 2). We use the common outlier definition from boxplots, i.e. all values with distance to the 1 ^st^ quartile (Q1) or 3 ^rd^ quartile (Q3) larger than 1.5 x the inter-quartile range Q3-Q1 are considered as outlier.

For evaluating the sensitivity with respect to exclusion, we perform an additional, secondary analysis on all synthetic data sets, regardless of similarity to the templates.

**Modification of differential abundance (DA) tests**

***Inflated runtime***

Data sets with a large number of features could lead to inflating runtimes for some statistical tests. If the runtime threshold for an individual test is exceeded for a specific data set, we split the data set, apply the test again on the subsets and afterwards merge the results. This split and merge procedure is repeated until the test runtime is below the threshold.

Here, we define the runtime threshold to be max. 1 hour per test. Then, in a worst case scenario, the 14 tests for the 10+1 data sets for each of the 38 template (5832 combinations) would need 244 days on a single core. Since we can conduct the tests on up to 64 cores, such a worst case scenario would still be manageable.

***Test failure***

If a DA test throws an error we omit the number of significant features and the overlap of significant features and report them as NA (not available) as it would occur in practice.

### Strategies to improve adherence to interventions {11c}

n/a

### Relevant concomitant care permitted or prohibited during the trial {11d}

n/a

### Provisions for post-trial care {30}

n/a

## Outcomes {12}

Aim 1: For each data set (experimental template and synthetic data) 43 data characteristics are calculated as described in Table 5. The difference of a data characteristic between a synthetic data and the corresponding data template is calculated as outcomes. For each feature which is closer to a normal distribution on the log-scale according to p-values of the Shapiro-Wilk test, we apply a log2-transformation to the respective characteristics prior to all analyses.

Principal component analysis (PCA) is then performed on the scaled data characteristics and a two-dimensional PCA plot is generated. An additional outcome is the Euclidean distance of a synthetic data set to its template in the first two principal component coordinates. An equivalence test will be conducted on the synthetic data sets for each template to check whether data properties are maintained in synthetic data on a summary level for all data characteristics.

Next, boxplots are generated, visualizing for each data characteristic how it varies between templates, between all simulation realizations, and how templates deviate from the corresponding synthetic data sets. Here, we again perform equivalence tests and also report median distances of a data characteristic between simulated and experimental data.

Aim 2: As primary outcome (aim 2a), for each experimental data template the average proportion of shared significant features across all synthetic data are calculated for each DA tool. For each data template, a barplot is generated as in Nearing et al. ^1^ to visually summarize how many of the 14 DA tools identified the same feature as significantly changed. Moreover, we try to validate the conclusions from Nearing et al. ^1^ made on this primary outcome. Overall, we extracted 13 conclusions and formulated the respective hypothesis as shown in Box 1.

Box 1. **Hypotheses investigating the overlap of identified features as aim 2a extracted as conclusions from Ref. 1 including the statistical analysis to be applied.**

We term the statistical analysis that estimates the proportion P of cases (e.g. the proportion of synthetic data sets) where the hypothesis is fulfilled as “Counting”. Depending on the stringency of the formulation in Ref. 1, we always define a question-specific threshold for confirmation and indicate the respective number of cases n for this evaluation in brackets. The asterisk * indicates that this number of cases might be reduced if exclusion criteria apply.

Hypothesis 1: For unfiltered data, the proportion of features jointly found as significant by limma voom TMM and limma voom TMMwsp but by less than 50% of the other methods, is larger than the overlap with more than 50% of the other methods.

Analysis: **Counting** (n=380*) with 95% threshold, i.e. the hypothesis is validated if the 95% CI is > 95%.

Hypothesis 2: For unfiltered data, the overlap of features jointly found as significant by limma voom TMM and limma voom TMMwsp with features found by Wilcoxon CLR is larger than the overlap with all other DA methods.

Analysis: **Counting** (n=380*) with 95% threshold.

Hypothesis 3: For unfiltered data, the Kolmogorov-Smirnov test statistic D when comparing the profile for Wilcoxon CLR and Wilcoxon is larger than for other pairs of methods on average.

Analysis: **Counting** (n=380*) with 95% threshold.

Hypothesis 4: For unfiltered data, MaAsLin2 and MaAsLin2-rare a more similar profile (larger test statistic D) than a randomly selected pair of methods.

Analysis: **Counting** (n=380*) with 95% threshold.

Hypothesis 5: For unfiltered data, ALDEx2 and ANCOM-BC identify more features that were also identified by all except 3 (i.e. 10 out of 13) other methods.

Analysis: **Counting** (n=380*) with 95% threshold.

Hypothesis 6: For unfiltered data, EdgeR and LEfSe identify a larger percentage of features that are not identified by any other tool, compared to the same percentage for all other methods.

Analysis: **Counting** (n=380*) with 95% threshold.

Hypothesis 7: For unfiltered data, for corncob, metagenomeSeq, and DESeq2, there are always multiple other methods (i.e. at least 2 out of 10 other DA methods) that have a more extreme consistency profile.

Analysis: **Counting** (n=380*) with 95% threshold. Mean consistency is used to assess the location of the consistency profile.

Hypothesis 8: The shape of the overlap profiles is mainly determined by the exp. data set and the DA method but only little of the fact whether data has been filtered.

Analysis: qq-Plots of the cumulative overlap profile filtered vs. non-filtered are closer to the diagonal than comparison of different DA methods and comparison of different exp. data templates. Quantification using the **mean of the Kolmogorov-Smirnov** test statistic, i.e. the average of the maximal absolute distance of the empirical cumulative density functions.

Hypothesis 9: For filtered data, for both limma voom approaches the proportion of identified features that are also identified by the majority of other tests is larger than for un-filtered data

Analysis: **Counting** (n=380*) with 95% threshold.

Hypothesis 10: For filtered data, the overlap profile of Wilcoxon CLR is bimodal.

Analysis: **Counting** (n=380*) with 95% threshold using bimodality index (R function BimodalIndex::bimodalIndex). Only datasets with at least 10 significant features when using Wilcoxon CLR will be considered.

Hypothesis 11: The proportion of features identified by all except one DA method is larger for prevalence-filtered data.

Analysis: **Counting** (n=380*) with 95% threshold.

Hypothesis 12: For filtered data, the consistency profiles of corncob, metagenomeSeq, and DESeq2 are more similar to the more extreme methods (as for Hypothesis 9 defined as the most extreme 2 profiles of other DA methods) than for unfiltered data.

Analysis: **Counting** (n=380*) with 95% threshold using the Kolmogorov-Smirnov test statistic of the two 2 ^nd^ most extreme left- and right shifted other profiles.

Hypothesis 13: For filtered data, ALDEx2 and ANCOM-BC identify more features that were also identified by all except 3 (i.e. 10 out of 13) other methods.

Analysis: **Counting** (n=380*) with 95% threshold.

As secondary outcome (aim 2b), the numbers and proportions of significant features across tools and data sets are considered. This outcome is reported and visualized in a heatmap with rows representing the data templates and columns the DA tools as in Nearing et al. ^1^ In total, two heatmaps, one for the experimental data and a second one for the simulated data, are generated. For the synthetic data sets, mean values from N=10 simulation realizations are calculated and plotted.

Moreover, we try to validate the outcomes from Nearing et al. ^1^ made on this secondary outcome. Overall, we extracted 14 conclusions about the number of identified features and formulated the respective hypothesis as summarized in Box 2.

Box 2. **Hypotheses investigating the proportion of identified features as aim 2b extracted as conclusions from Ref. 1 including the statistical analysis to be applied.**

We term the statistical analysis that estimates the proportion P of cases (e.g. the proportion of synthetic data sets) where the hypothesis is fulfilled as “Counting”. Depending on the stringency of the formulation in Ref. 1, we always define a question-specific threshold for confirmation and indicate the respective number of cases n for this evaluation in brackets. The asterisk * indicates that this number of cases might be reduced if exclusion criteria apply.

Hypothesis1: For filtered and unfiltered data, the percentage of significant features identified by each DA method varies widely across data sets.

Analysis: **Histograms** of the range (= max – min) of the percentage values 1) for all data sets, and 2) for all DA methods when applied to data from each data template

Hypothesis 2: For filtered and unfiltered data, rankings of the DA methods with respect to the proportion of identified features depend on the data template.

Analysis: **2-way ANOVA** of the rank-transformed proportions using DA method and template as grouping variables indicate significant interaction effects between both variables.

Hypothesis 3: Rankings of the DA methods with respect to the proportion of identified features depend stronger on the data template in unfiltered data than in filtered data sets.

Analysis: **2-way ANOVA** of the ranks using DA method and template as grouping variables indicate a more significant interaction effects between both effects in unfiltered data (compared to respective analysis for unfiltered data, see preceding hypothesis).

Hypothesis 4: In unfiltered data, either limma voom TMMwsp, limma voom TMM, Wilcoxon, or LEfSe identify the largest proportion of significant features.

Analysis: **Counting** ( n=380*). Since in the original analysis, the observation was seen in all analysis, we classify the hypothesis as confirmed, if true in >95% of cases.

Hypothesis 5: For unfiltered data, there are data sets, where edgeR identifies the largest proportion of significant features.

Analysis: **Counting** (n=380*) with >0 threshold.

Hypothesis 6: For unfiltered data, Limma voom TMMwsp identifies the largest proportion of features in the Human-HIV data set.

Analysis: **Counting** (n=380*) in all synthetic data sets generated for this template with >95% threshold.

Hypothesis 7: For unfiltered data, there are data sets, where both limma voom methods identify more than 99% of features as significant

Analysis: **Counting** (n=380*) with >0 threshold.

Hypothesis 8: For unfiltered data, there are data sets, where Wilcoxon CLR identifies more than 90% of features as significant

Analysis: **Counting** (n=380*) with >0 threshold.

Hypothesis 9: For unfiltered data, there are data sets, where LEfSe identifies more than 99% of features as significant

Analysis: **Counting** (n=380*) with >0 threshold.

Hypothesis 10: In unfiltered data, either ALDEx2 or ANCOM-BC identify the fewest significant features.

Analysis: **Counting** (n=380*) with >95% threshold.

Hypothesis 11: In unfiltered data, ALDEx2, ANCOM-BC and corncob do not identify significantly more features than the most conservative tests.

Analysis: **Counting** (n=380*) whether DA method is within the three most conservative ones (with >95% threshold).

Hypothesis 12: No tool (except ALDEx2) identifies a smaller number of features for unfiltered data (compared to filtered data).

Analysis: **Counting** with >95% threshold.

Hypothesis 13: For filtered and unfiltered data, ANCOM-BC identifies the least significant features in total, i.e. when summing ranks of DA methods over all 38 templates.

Analysis: **Counting** (n=10) whether the ranksum over 38* single synthetic data simulations (randomly drawn from N=10) of all other DA methods satisfies the hypothesis (>95% threshold without confidence intervals).

Hypothesis 14: For filtered data, there is no method other than EdgeR, LEfSe, limma voom, or Wilcoxon that identifies the largest number of significant features in total, i.e. when considering ranks of DA methods over all 38 templates.

Analysis: **Counting** (n=10) whether the ranksum over 38* single synthetic data simulations (randomly drawn from N=10) of all other DA methods satisfies the hypothesis (<5% threshold without confidence intervals).

### Participant timeline {13}

n/a

### Sample size {14}

The used 38 experimental data sets and DA tests are defined by the reference study that is being validated. In our study, we can therefore only choose the number of synthetic data sets per data template reasonably. Since there is no pilot study for the outcomes obtained from synthetic data that can be used for sample size calculations, and because a large number of outcomes are considered, the number of simulated data sets for one template was chosen to be a) feasible in terms of run time and b) should be large enough to enable valid conclusions. Based on both aspects, we decided to simulate N=10 synthetic data sets for each experimental data template, i.e., 380 synthetic data sets for each simulation tool.

For the one-sample equivalence tests with significance level 5% conducted for aim 1, N=10 synthetic data sets have a power of 89,75% to reject both the null hypothesis that the data characteristic is below -1SD and above +1SD and thereby favor the alternative hypothesis that the characteristic is equivalent to the reference value computed for the experimental data template. For this computation, we presumed that the expected mean (i.e. the bias of a characteristic in simulated data) is 0.5 SD, the standard deviation within synthetic data is 0.5 SD. Here, all specifications were made in units SD that refers to the standard deviation over all exp. templates. Assuming a smaller bias or a smaller variability for the synthetic data increases the expected power. These calculation has been conducted with Nquery version 8.7.2.0, equivalence test for one mean (MOE4-1).

For the proportions P of fulfilled hypothesis that were estimated for addressing most hypotheses in aim 2, 95% Clopper-Pearson confidence intervals (R-package DescTools) are calculated. As an example, n=380 independent samples leads to P = 0.026, 95%-CI = [0.013, 0.047] if for 10 out of 380 synthetic data sets the tested hypothesis is violated. This sample size is available for 20/27 hypotheses. However, it should be noted that such sample size considerations are limited by the fact that the data characteristics and results for synthetic data sets from a template are likely to be similar to each other and it is therefore not permissible to consider all samples as independent.

### Recruitment {15}

n/a

### Assignment of interventions: allocation

### Sequence generation {16a}

The intervention and the comparator can be evaluated for all data sets and the order of the computations has no impact. Therefore, random allocation and sequence generation is not required.

### Concealment mechanism {16b}

n/a

### Implementation {16c}

n/a

### Assignment of interventions: Blinding

### Who will be blinded {17a}

No blinding procedure will be applied because we see no risk of bias by a non-blinded calculation of data characteristics and statistical tests.

### Procedure for unblinding if needed {17b}

n/a: No blinding performed.

## Data collection and management

### Plans for assessment and collection of outcomes {18a}

The analyses will be conducted by two experienced Statisticians/Bioinformaticians (E.K., C.K.) both with > 5yrs experience with differential abundance analyses. All statistical tests will adhere to the methodology outlined by Nearing et al. ^1^ To do so we chose the same configurations of the statistical methods as implemented in the code from Nearing et al. ^1^ for running the statistical tests, provided on Github ( https://github.com/nearinj/Comparison_of_DA_microbiome_methods). This strategy ensures comparability between the outcomes of the reference study and our validation study.

### Plans to promote participant retention and complete follow-up {18b}

n/a

### Data management {19}

The 38 experimental data sets were downloaded from https://figshare.com/articles/dataset/16S_rRNA_Microbiome_Datasets/14531724 on February 9, 2024. There, Nearing et al. ^1^ made the data sets from their study available, therefore we incorporate the exact same data sets. We keep a local copy of this data in our Fredato research data management system https://nxc-fredato.imbi.uni-freiburg.de until 31.12.2030 and make it available if the original data is not available in the current version any more and if this does not violate legal, data protection, or copyright regulations. Generated data, analysis scripts, results and supplemental information to this study will also be stored in the Fredato research data management. In case of unexpected technical limitations, we will make data, analysis scripts and supplemental information available via https://figshare.com.

### Confidentiality {27}

n/a: The experimental data is publicly available. The data generated in this project is not subject to data protection regulations.

### Plans for collection, laboratory evaluation and storage of biological specimens for genetic or molecular analysis in this trial/future use {33}

n/a

## Statistical methods {20}

### Data monitoring committee {21a}

n/a: The data has already been collected and is publicly available.

### Statistical methods for primary and secondary outcomes {20a}

Aim 1: For each data set (experimental and synthetic) a set of 43 data characteristics is calculated. All data characteristics are defined as a single number. These calculations are described in more detail in Table 5. As N=10 simulation realizations are generated, there will be 10 values for each data characteristic per experimental data template. For the primary outcome a PCA plot based on the scaled data characteristics is generated. Moreover, equivalence tests are applied to all 43 data characteristics as well as to the Euclidean distance in the two principal component coordinates as describe above (section “Interventions”). Based on these equivalence tests, we test a single hypothesis (equivalence of synthetic and experimental data) which is fulfilled in strict terms only when all equivalence tests are significant. We therefore do not have to control the probability of a single false positive test (i.e. the so-called family-wise error rate). Therefore, multiple testing aspects do not apply these tests.

Aim 2: All DA methods will be applied to experimental and synthetic data adhering to the methodology in Nearing et al. ^1^ as described in the methods section of the paper. Significant features will be identified using a 0.05 threshold for the multiple testing adjusted p-values (Benjamini-Hochberg).

For the primary outcome, it is determined how many tests jointly identify features to be significant for each data set. After visualization, the 13 hypotheses extracted from ^1^ will be investigated using the statistical analyses summarized in Box 1. Estimates of the target values and the respective 95% confidence intervals are used to validate the hypotheses because these values are easier to interpret than p-values and because the significance of the p-values is strongly determined by the number of cases.

For the secondary outcome, the number and proportion of significant features is extracted for each data set and test individually. After visualization, the 14 hypotheses extracted from ^1^ will be investigated using the statistical analyses summarized in Box 2.

Confidence intervals for the estimated proportions of cases where the hypothesis is fulfilled will be calculated as exact intervals, i.e. using Clopper-Pearson intervals (R-package DescTools). If the analyzed cases are statistically dependent, we compute hierarchical bootstrap confidence intervals by first drawing with replacement templates, and then synthetic data sets within each template.

These analyses are conducted for unfiltered data and for filtered data. As in, ^1^ filtered means that features found in fewer than 10% of samples are removed. Moreover, to analyze the sensitivity of our outcomes with respect to our exclusion criteria, filtered and unfiltered data are also analyzed without applying criteria that exclude non-realistic simulations.

In case we find different results for the simulated data for some hypotheses, we will analyze the association of the mismatch in the outcome with the mismatch of data characteristics to identify data characteristics that could be responsible for the disagreement. To ensure independence of the scales, we will perform these analyses after rank transformations. We will use univariate analyses (i.e. Spearman correlation) as well as a forward selection with a 5% cut-off criterion for p-values.

### Interim analyses {21b}

n/a: There will be no interim analyses in this study.

### Methods for additional analyses (e.g. subgroup analyses) {20b}

n/a: In this study there will be no subgroup analyses.

### Methods in analysis to handle protocol non-adherence and any statistical methods to handle missing data {20c}

n/a: In this study there is no missing data.

### Plans to give access to the full protocol, participant level-data and statistical code {31c}

Generated data, analysis scripts and supplemental information to this study will also be stored in the Fredato research data management. In case of unexpected technical limitations, we will make data, analysis scripts and supplemental information available via https://figshare.com.

## Timeline

There is no timeline for data collection, as the study is based on existing data. Conducting the study does not depend on any clinical parameters. The anticipated timeline for completing the study is 3 to 4 months.

## Oversight and monitoring

### Composition of the coordinating centre and trial steering committee {5d}

n/a

### Composition of the data monitoring committee, its role and reporting structure {21a}

n/a

### Adverse event reporting and harms {22}

n/a

### Ancillary and post-trial care {30}

n/a

### Frequency and plans for auditing trial conduct {23}

n/a

### Plans for communicating important protocol amendments to relevant parties (e.g. trial participants, ethical committees) {25}

n/a

## Dissemination policy {31a}

Public access of the generated data, analysis scripts, results and supplemental information is granted as indicated above on our Fredato research data management system https://nxc-fredato.imbi.uni-freiburg.de. The results will be published in a peer-reviewed scientific journal, preferably in the same journal as this protocol.

## Discussion

Synthetic data are a valuable tool in benchmark studies as it allows for controlled manipulation of data properties and evaluations based on the underlying known truths. We utilize this potential to validate previous findings derived on experimental data exclusively. However, first it is critical to determine the extent to which synthetic data can faithfully reflect real experimental data. It may necessitate adjustments to simulation tools to ensure their capability to accurately mimic experimental conditions. Consequently, this study not only investigates the potential limitations of synthetic data in validating experimental outcomes but also sheds light on the general capacity of simulation tools to faithfully represent real-world data. Since unsuccessful validation indicates that the previously published findings do not generalize to synthetic data, we try to identify responsible inadequacies in the simulation tools. However, this aspect is limited to the data characteristics of our study and there might be additional data properties that require consideration to explain a possible mismatch. Finally, this benchmark study employs rigorous statistical methodology and, for the first time, publishes a study protocol in advance, adhering to established protocol guidelines. Moreover, it is the first benchmark study that primarily focus on validation of the results of a previous study.

## Abbreviations

ANCOM: Analysis of compositions of microbiomes with bias correction, a DA method
ANOVA: analysis of variance
DA: differential abundance
Fredato: Freiburg research data management tool
LEfSe: Linear discriminant analysis Effect Size, a DA method
MaAsLin: Microbiome Multivariable Association with Linear Models, a DA method
metaSPARSim: acronym for “a sparse count matrix simulator intended for usage in development of 16S rDNA-seq metagenomic data processing pipelines” ^18^
p _KS_
: p-values of a Kolmogorov-Smirnov test
PCA: principal component analysis
R: a script-based statistical programming language
sparseDOSSA2: abbreviation for “Sparse Data Observations for Simulating Synthetic Abundance”, a simulation tool for microbial count data based on a hierarchical model ^19^
TMM: trimmed mean of M values, an approach for data normalization
TMMwsp: TMM with singleton pairing, an approach for data normalization

## Declarations

## Authors’ contributions {31b}

E.K. and C.K. equally contributed to the design and development of the study.

## Ethics and consent {24}

Ethical approval and consent were not required

## Consent for publication {32}

n/a

## Authors’ information (optional)

E.K.: Statistical data analyst and PhD student in the lab of Dr. Clemens Kreutz. Research topics cover robust data analysis of high throughput data with a specification for microbiome sequencing data.

C.K.: Group Leader of the lab for Methods in Systems Biomedicine at the Institute for Medical Biometry and Statistics of University Medical Center of Freiburg. Research topics cover mathematic modelling for systems biomedicine and neutral benchmark studies.

Both authors have neither been involved in the development of any of the applied simulation and DA methods nor in the reference study. ^1^

## Study status

## Data Availability

No data are associated with this article.
