# Supplementary material for: Computational Study Protocol: Leveraging Synthetic Data to Validate a Benchmark Study for Differential Abundance Tests for 16S Microbiome Sequencing Data
Source: F1000Res. 2025 Jan 2;13:1180. Originally published 2024 Oct 9. [Version 2] doi: 10.12688/f1000research.155230.2 (PMC11757917; doi:10.12688/f1000research.155230.2)
Supplement: Peer review history

# Reviewer report: Reviewer response for version 2

**DOI:** 10.5256/f1000research.176118.r355372

**Published:** 2025-01-23

**Authors:** Jacob T. Nearing

**Affiliations:** 1 Biostatistics, Harvard T.H. Chan School of Public Health, Boston, MA, USA

The authors have addressed all my comments in their revisions and clarified the manuscript's goals and objectives.  

 I have no further comments to provide.

Is the study design appropriate for the research question?

Partly

Is the rationale for, and objectives of, the study clearly described?

Partly

Are sufficient details of the methods provided to allow replication by others?

Partly

Are the datasets clearly presented in a useable and accessible format?

Yes

Reviewer Expertise:

Bioinformatics, microbiome

I confirm that I have read this submission and believe that I have an appropriate level of expertise to confirm that it is of an acceptable scientific standard.

# Reviewer report: Reviewer response for version 2

**DOI:** 10.5256/f1000research.176118.r355371

**Published:** 2025-01-21

**Authors:** Leo Lahti, Juho Pelto

**Affiliations:** 1 University of Turku, Turku, Finland; 2 Department of Computing, University of Turku, Turku, Finland

The authors have responded to our comments satisfactorily. Most importantly, the rationale for and the objectives of the study are now more clearly expressed.

 However, in case that metaSPARSim and sparseDOSSA2 cannot produce (even after adjustments for sparsity and effect sizes) data sets that resemble the original 38 real data sets closely enough, it remains a limitation of the study that no other simulation tools are being used.

 While we understand that adding other simulation tools to the study would increase the run-time and complexity of the study, testing other tools could be considered at least conditionally, i.e. in case that metaSPARSim and sparseDOSSA2 cannot produce proper data.

Is the study design appropriate for the research question?

Partly

Is the rationale for, and objectives of, the study clearly described?

Partly

Are sufficient details of the methods provided to allow replication by others?

Partly

Are the datasets clearly presented in a useable and accessible format?

Yes

Reviewer Expertise:

biostatistics

We confirm that we have read this submission and believe that we have an appropriate level of expertise to confirm that it is of an acceptable scientific standard.

# Reviewer report: Reviewer response for version 1

**DOI:** 10.5256/f1000research.170374.r340427

**Published:** 2024-12-05

**Authors:** Jacob T. Nearing

**Affiliations:** 1 Biostatistics, Harvard T.H. Chan School of Public Health, Boston, MA, USA

In this protocol Kohnert and Kreutz design a study to assess microbiome differential abundance methods by replicating Nearing et al., 2022 using synthetic datasets generated from the original data using metaSPARSim and sparseDOSSA2. They intend to determine the similarity between the synthetic data generated and their underlying true datasets using 43 different data characteristics. Moreover, they hope to identify whether there are any driving characteristics in synthetic data that would result in discordant conclusions with those achieved by using the real datasets they are based upon. The authors identify that their protocol falls within SPIRIT guidelines and openly report all changes that were made to their report over its initial creation until now. Overall, the study is of interest to microbiome research, however, some areas require clarification, and the overall presentation of the protocol could be improved as major steps in some cases were not presented in order.

Major comments:

 Well I appreciate the authors adherence to the SPIRIT guidelines it made the ordering of the steps in this protocol hard to follow as these guidelines do not fully align with computational benchmarking studies. For example, the under the section “ **Modification by adjusting the proportions of zeros and effect sizes” **it was mentioned that the groups would be simulated separately, however, the exact simulation procedure (and parameters for metaSPARSim and sparseDOSSA2) was not fully discussed prior to this. Additional information on the code and parameters used in this simulation software (including version # should be included).

 I suspect the authors may have difficulty fully recapturing all the true effect size differences between groups within the same study using synthetic simulated data. Given this I appreciated the in-depth discussion they gave, and the illustration presented in figure 2. However, I do believe that the study could benefit from treating equivalence as a sliding scale rather than a discreate category for each synthetic dataset based on an equivalence test for each data characteristic. It is possible that being equivalent in one characteristic will result in a much larger range than another and this should be taken into consideration during further data analysis. As an aside to this the authors should consider cases where standard deviations may become so large that almost any dataset may pass the equivalence test that it is subjected to (i.e. in figure 1 what happens if the upper and lower bounds cover almost the entire possible range of that data characteristic). In this case it may be reasonable to look at how many times a synthetic dataset from an unrelated template passes an equivalence test. (i.e. how many times does an unrelated dataset that shows very different DA results show equivalence and in what data characteristics does this occur in?). 

 In figure 2 and in the section " **Exclusion of simulations for a specific data template based on deviating data properties" **it is suggested that some data characteristics may be too sensitive to include during equivalency testing, however, there is no guidelines to the best of my interpretation of the protocol to determine when a data characteristic would be too sensitive that it must be disregarded. 

Minor comments:

 Some tools included within this study may have undergone significant updates since the initial publication of Nearing et al., 2022. This should be considered in the analysis as even slightly differences may give rise to discordant results. As an aside to this the authors may want to consider including more recently developed methods in their study such as LOCOM, ANCOM-BC2 or radEmu. This would benefit the microbiome community. 

 In some cases, the hypothesis they define for aims 1 and 2 should be reviewed for clarity. I have listed some examples below.  

 The authors mention ANCOM-BC however, this DA tool was not considering in Nearing et al., 2022. It is possible that this was typo, and the authors were suggesting ANCOM-II, however, it is important to clarify this as ANCOM-II and ANCOM-BC are highly different methods. 

 Hypothesis 8 in aim 1 should be adjusted as the shapes were similar except for the limma voom tools.

 Hypothesis 4 in aim 2 should specify what Wilcoxon method is being referred to.

 Hypothesis 9 in aim 2 suggests that in the original study LEFSE had results where more than 99% of features in a dataset were significant, however, I do think this was found in Nearing et al., 2022. Although it was identified to be on the more sensitive side of methods tested.

Is the study design appropriate for the research question?

Partly

Is the rationale for, and objectives of, the study clearly described?

Partly

Are sufficient details of the methods provided to allow replication by others?

Partly

Are the datasets clearly presented in a useable and accessible format?

Yes

Reviewer Expertise:

Bioinformatics, microbiome,

I confirm that I have read this submission and believe that I have an appropriate level of expertise to confirm that it is of an acceptable scientific standard, however I have significant reservations, as outlined above.

# Response

**Published:** 2024-12-17

**Authors:** Eva Kohnert

**Affiliations:** Medical Center-University of Freiburg, Freiburg, Baden-Württemberg, Germany

**Major comments:**

*Well I appreciate the authors adherence to the SPIRIT guidelines it made the ordering of the steps in this protocol hard to follow as these guidelines do not fully align with computational benchmarking studies. For example, the under the section “Modification by adjusting the proportions of zeros and effect sizes” it was mentioned that the groups would be simulated separately, however, the exact simulation procedure (and parameters for metaSPARSim and sparseDOSSA2) was not fully discussed prior to this. Additional information on the code and parameters used in this simulation software (including version # should be included).*

 In the revised version of our study protocol, we describe in more detail, how the simulated data is generated. We also added version numbers for the two simulation packages. All package versions will be provided when the study is accomplished. We also added a reference to paragraph “Modification by adjusting the proportions of zeros and effect sizes” in section 11b where it is described, how the simulation procedure is changed to adapt the effect size.

*I suspect the authors may have difficulty fully recapturing all the true effect size differences between groups within the same study using synthetic simulated data. Given this I appreciated the in-depth discussion they gave, and the illustration presented in figure 2. However, I do believe that the study could benefit from treating equivalence as a sliding scale rather than a discreate category for each synthetic dataset based on an equivalence test for each data characteristic. It is possible that being equivalent in one characteristic will result in a much larger range than another and this should be taken into consideration during further data analysis. As an aside to this the authors should consider cases where standard deviations may become so large that almost any dataset may pass the equivalence test that it is subjected to (i.e. in figure 1 what happens if the upper and lower bounds cover almost the entire possible range of that data characteristic). In this case it may be reasonable to look at how many times a synthetic dataset from an unrelated template passes an equivalence test. (i.e. how many times does an unrelated dataset that shows very different DA results show equivalence and in what data characteristics does this occur in?).*

 To address these concerns, we first want to emphasize that each data characteristic (DC) is computed independently for both experimental and simulated count matrices. The scales of different DCs are inherently incomparable; for instance, the proportion of zeros ranges between 0 and 1, while the number of features varies from 327 to 59,736. Thus, the vertical axis in Fig. X is in arbitrary units and +/- 1 SD always cover approx. 68% of the points from the exp. templates. Using the SD over the DCs computed from all exp. templates for defining the equivalence region can be seen as a normalization of the DCs to make it comparable.

 Our current equivalence testing yields a binary outcome (equivalent or not) to identify and exclude unrealistic simulations. We acknowledge the reviewer's suggestion to treat equivalence as a continuous outcome. However, implementing a sliding scale approach does not provide a final decision and presents challenges due to the diverse scales and distributions of DCs and we have to define a strategy for classification beforehand.

 Deciding based on how many times a synthetic dataset from an unrelated template passes an equivalence test could behave unintendedly if all synthetic data sets are unrealistic. Thus, we see no clear benefit and a further cutoff has to be defined. Nevertheless, if the reviewer prefers such an option, we are open to implement this (e.g. by defining the equivalence region from the 10% and 90% quantiles of the DC difference to unrelated templates).

 In response to all reviewers’ feedback, we have refined our set of DCs by eliminating redundancies and incorporating measures of alpha and beta diversity. To further address the reviewer's concerns, we will include a visualization by plotting the number of non-equivalent characteristics against the size of the equivalence region for comparing simulated data with the respective templates and comparing with unrelated templates. This will illustrate the relationship between equivalence thresholds and the proportion of DCs deemed equivalent / non-equivalent, offering deeper insight into the sensitivity of our equivalence testing procedure.

 We hope that these clarifications and proposed modifications adequately address the reviewer's concerns and enhance our study design.

*In figure 2 and in the section "Exclusion of simulations for a specific data template based on deviating data properties" it is suggested that some data characteristics may be too sensitive to include during equivalency testing, however, there is no guidelines to the best of my interpretation of the protocol to determine when a data characteristic would be too sensitive that it must be disregarded.*

 We apologize for not providing a clear definition earlier. We now define data characteristics more precisely: we will only consider those that pass equivalence tests for at least 50% of the templates.

**Minor comments:**

*Some tools included within this study may have undergone significant updates since the initial publication of Nearing et al., 2022. This should be considered in the analysis as even slightly differences may give rise to discordant results. As an aside to this the authors may want to consider including more recently developed methods in their study such as LOCOM, ANCOM-BC2 or radEmu. This would benefit the microbiome community.*

 As written in our study protocol (section 9), we’ll apply the latest versions of the DA methods since this seems mostly valuable. We are aware, that updates of the DA methods may be responsible for failed validations. However, we see assessment and confirmation using the most recent tools as more important than preventing the risk that disagreement might originate from improved DA tools.

 Since a key feature of our study is to strictly perform validations following a strict methodology, we do not want to mix validation with evaluation of other tests. This will be investigated in a subsequent study where we focus on sensitivities and specificities. In the revised version of the protocol, we mention this aspect and better emphasize our overall strategy.

 We emphasize this strategy in the revised version of the study protocol at several points and use wordings like “validation with the most recent implementations/versions”.

*In some cases, the hypothesis they define for aims 1 and 2 should be reviewed for clarity. I have listed some examples below.*

*The authors mention ANCOM-BC however, this DA tool was not considering in Nearing et al., 2022. It is possible that this was typo, and the authors were suggesting ANCOM-II, however, it is important to clarify this as ANCOM-II and ANCOM-BC are highly different methods.*

 We thank the reviewer for this note. Upon reflection, we recognize that we indeed planned to use a different ANCOM method than the one employed in Nearing et al. They utilized a custom script downloaded “on May 11, 2020, from the FrederickHuangLin/ANCOM" repository on GitHub.  We will use this script for our analysis to be as consistent as possible with the reference study.

*Hypothesis 8 in aim 1 should be adjusted as the shapes were similar except for the limma voom tools.*

 Thank you for pointing this out. We changed the hypothesis accordingly.

*Hypothesis 4 in aim 2 should specify what Wilcoxon method is being referred to.*

 We checked all hypothesis and clearly indicated which method is being referred to.

*Hypothesis 9 in aim 2 suggests that in the original study LEFSE had results where more than 99% of features in a dataset were significant, however, I do think this was found in Nearing et al., 2022. Although it was identified to be on the more sensitive side of methods tested.*

 Thank you for pointing that out. We changed the hypothesis to be in line with the results from the reference paper.

*Is the rationale for, and objectives of, the study clearly described?*

*Partly*

 We acknowledge that the rationale and objectives of the study may not have been communicated clearly. To clarify this, we changed the abstract. To summarize, this study is essentially a validation study, focusing on evaluating the feasibility of using synthetic data to resemble experimental data and whether we replicate findings from the reference study based on multiple simulated data sets. Additionally, it is a showcase how to incorporate study protocols in computational studies and to rigorously plan a benchmark study before actually conducting it. This study serves as groundwork for a subsequent benchmark study, which will include a broader range of data sets for performance testing.

*Is the study design appropriate for the research question?*

*Partly*

 We agree that simulated data has limitations in fully validating previous findings due to inherent differences from experimental data. To address this, we calculate a comprehensive set of data characteristics, perform equivalence tests, and select only simulated data that does not show significant disagreement with experimental data. In our view, this approach is the best we can do to minimize the differences and ensure comparability between simulated and experimental datasets. Moreover, applying the presented equivalence testing procedure represents a novel methodological improvement compared to existing approaches.

*Are sufficient details of the methods provided to allow replication by others?*

*Partly*

 In the revised version we better explain especially that data generation mechanism, by including more details for the simulation process. All data sets, experimental and generated, as well as all source code to run the study will be made available once the paper describing the results will undergo review process.

*Are the datasets clearly presented in a useable and accessible format?*

*Yes*

# Reviewer report: Reviewer response for version 1

**DOI:** 10.5256/f1000research.170374.r340429

**Published:** 2024-11-27

**Authors:** Leo Lahti, Juho Pelto

**Affiliations:** 1 University of Turku, Turku, Finland; 2 Department of Computing, University of Turku, Turku, Finland

This manuscript suggests a (computational) protocol for validating differential abundance tests. The main question is whether synthetic data, generated to mimic experimental data, can validate the findings from differential abundance tests for 16S microbiome sequencing data. Specifically, the study aims to assess how well synthetic datasets replicate the characteristics of real data and whether the conclusions from real data on the performance of DA tests can be confirmed with synthetic data. In order to evaluate such protocol, the authors created synthetic data that would resemble the 16S rRNA gene sequencing data in the 38 datasets employed in the study by Nearing et al. (2022). They mimic real data with synthetic data using metaSPARSim and sparseDOSSA2 with specific adjustments. They use 43 metrics to evaluate the correspondence between the synthetic and real data. The study complies with the SPIRIT guidelines for clinical trial protocols. Second, the datasets that resemble the original data sufficiently well were used to replicate results of differential abundance analysis (DAA) method comparison by Nearing et al. The language is clear but the protocol has a vast number of steps, making it hard to separate the key contributions and performance assessment from the routine protocol checkpoints.

 Major remarks:

 1) In addition to using metaSPARSim and sparseDOSSA2, consider including more recent methods such as SimMSeq (Yang and Chen, 2022) and SIMBA (Wirbel et al., 2024).

 2) The authors use 43 metrics to assess the correspondence between synthetic and real data. The number of metrics seems quite high, and it may thus be difficult describe these resemblances. Would it be possible to identify a smaller number of key metrics that would grasp the most essential aspects of the data?

 3) The motivation of Aim 2 seems unclear. The authors state in the introduction, synthetic data can be used to evaluate the correctness of DAA results. However, the authors suggest to use synthetic data to replicate the results Nearing et al. (Aim 2). Why synthetic data is not used to directly evaluate the correctness of the DAA results? Replication of Nearing’s results to evaluate the validity of the synthetic data seems a rather indirect means to assess the performance of the DAA methods.

 4) The study is about 16S (according to the title and descriptions) but Table 2, Aim 1 discusses metagenome simulation tools. The 16S and metagenome are different approaches for characterizing microbial communities, although there are some overlaps. It would require further clarification why a main Table switches to metagenomics in a 16S paper.

 5) Availability of materials: the study is based on an open data set that is available. Availability of the source code for the experiments and results would be critical for this kind of study. I could not locate these from the manuscript. Instead, there is a text "Generated data, analysis scripts, results and supplemental information to this study will also be stored in the Fredato research data management. In case of unexpected technical limitations, we will make data, analysis scripts and supplemental information available via https://figshare.com". These materials should be made available already at the review stage, otherwise a full review is not possible.

 Minor notes:

 - Real data sets are inherently different from synthetic ones. If the underlying data distributions differ significantly between synthetic and experimental datasets, the statistical tests may yield misleading results. Some discussion could be added on this and other potential limitations of using synthetic data for methods validation. 

 - Possibly out of scope for the protocol study but in case all the compared data generation methods fail to produce realistic data can the authors consider suggesting their own solution to generate realistic 16S data or expand discussion on this? 

 - Could you consider using other similar measures to PCA, for instance, principal coordinate analysis based on different dissimilarity metrics (e.g. Jaccard and Bray-Curtis distances) to evaluate the robustness of the ordination comparison?

 - Table 2: " stasticial analyses" -> "statistical analyses"?

Is the study design appropriate for the research question?

Partly

Is the rationale for, and objectives of, the study clearly described?

Partly

Are sufficient details of the methods provided to allow replication by others?

Partly

Are the datasets clearly presented in a useable and accessible format?

Yes

Reviewer Expertise:

bioinformatics, microbiome, data analysis

We confirm that we have read this submission and believe that we have an appropriate level of expertise to confirm that it is of an acceptable scientific standard, however we have significant reservations, as outlined above.

## References

[1] : A comprehensive evaluation of microbial differential abundance analysis methods: current status and potential solutions. *Microbiome* .2022;10(1) : 10.1186/s40168-022-01320-0 130 10.1186/s40168-022-01320-0 35986393 PMC9392415

[2] : A realistic benchmark for differential abundance testing and confounder adjustment in human microbiome studies. *Genome Biol* .2024;25(1) : 10.1186/s13059-024-03390-9 247 10.1186/s13059-024-03390-9 39322959 PMC11423519

# Response

**Published:** 2024-12-17

**Authors:** Eva Kohnert

**Affiliations:** Medical Center-University of Freiburg, Freiburg, Baden-Württemberg, Germany

General remark:

*The language is clear but the protocol has a vast number of steps, making it hard to separate the key contributions and performance assessment from the routine protocol checkpoints.*

 We appreciate the reviewer’s feedback regarding the structure and clarity of the protocol. Since this is our first attempt at drafting a study protocol for a computational benchmark study, we adhered strictly to the SPIRIT guidelines. This allowed us to explore the applicability of these guidelines, originally designed for clinical research, and how they could be effectively adapted to computational studies in the future. While we acknowledge that this approach may have resulted in a protocol with numerous steps, we believe this provides a comprehensive foundation for reproducibility and transparency in computational benchmarking.

 For future benchmark studies, we plan to refine the protocol structure, focusing on clearly delineating key contributions and performance assessments from routine protocol checkpoints.

**Major remarks:**

*1) In addition to using metaSPARSim and sparseDOSSA2, consider including more recent methods such as SimMSeq (Yang and Chen, 2022) and SIMBA (Wirbel et al., 2024).*

 We appreciate the reviewer’s suggestion to consider additional simulation tools such as SimMSeq and SIMBA. While we recognize the potential value of these tools, there are several reasons why we chose not to include additional simulation methods in this study:

 First, the current study is already highly complex, incorporating two simulation tools, comprehensive characterization of various data sets, multiple inclusion/exclusion criteria and extensive testing. Adding further tools would further increase the study’s complexity, potentially detracting from the clarity of our findings.

 Secondly, as stated in the protocol the computational effects are huge. Therefore, we defined a runtime threshold of max. 1 hour per differential abundance test. We are calculating 14 differential abundance tests across 10 + 1 (simulation + experimental template) datasets for each of the 38 templates and for unfiltered and filtered data, leading to a total of 11,704 combinations. In a worst case scenario, the runtime on a single core would already require approximately 488 days per simulation tool only for conducting DA analyses. Adding additional simulation tools would further inflate runtime, particularly given the additional time required to calibrate the simulators and generate synthetic datasets.

 Lastly, one of the key aims of this study is to introduce and emphasize the importance of formulating a study protocol specifically for computational research. This is the first attempt to define a structured protocol for such studies, and while it may not yet represent the most refined version, we think it is an important step towards robust and transparent computational benchmark studies. Developing this protocol required substantial effort, and we believe that establishing and demonstrating the concept of a protocol in computational studies is as important as the study results themselves.

 In the new version of the protocol, we briefly mention these aspects.

*2) The authors use 43 metrics to assess the correspondence between synthetic and real data. The number of metrics seems quite high, and it may thus be difficult describe these resemblances. Would it be possible to identify a smaller number of key metrics that would grasp the most essential aspects of the data?*

 We agree with the reviewer that a smaller set of metrics providing independent information would be beneficial. However, we aim to retain characteristics that might highlight discrepancies between simulated and experimental data and help explain potential disagreements in DAA outcomes. Consequently, we decided to eliminate metrics that are nearly redundant by iteratively removing those with a rank correlation ≥ 0.95 and adjusted our study protocol accordingly.

*3) The motivation of Aim 2 seems unclear. The authors state in the introduction, synthetic data can be used to evaluate the correctness of DAA results. However, the authors suggest to use synthetic data to replicate the results Nearing et al. (Aim 2). Why synthetic data is not used to directly evaluate the correctness of the DAA results? Replication of Nearing’s results to evaluate the validity of the synthetic data seems a rather indirect means to assess the performance of the DAA methods.*

 Given this feedback, we realized that the motivation of Aim 2 has not been communicated clearly. This study originated as part of the planning process for a more classical benchmark study using the underlying truth of synthetic data for assessing DA methods. While designing the benchmark study, we recognized the need to first assess the feasibility of generating synthetic data that realistically resemble all characteristics of experimental data, to ensure the validity of conclusions drawn from simulated data. This led us to develop the presented validation study with primary goal to compare the results based on synthetic data to those from the reference study. This study is going to be followed by a subsequent benchmark study, in which the known truth in the simulated data sets will be used for performance testing, and the dependence on characteristics such as effect size, sample size etc. will be systematically evaluated.

 We addressed this issue by updating the abstract and the description in section “study setting”, making it clearer that the focus of the presented study is in validating previous findings, and not in trying to estimate sensitivities and specificities which are planned as a subsequent project. The methodological advancement lies not only in incorporating synthetic data for validation but also in the development of a comprehensive study protocol.

*4) The study is about 16S (according to the title and descriptions) but Table 2, Aim 1 discusses metagenome simulation tools. The 16S and metagenome are different approaches for characterizing microbial communities, although there are some overlaps. It would require further clarification why a main Table switches to metagenomics in a 16S paper.*

 Thank you for pointing out that we were not precise in our terminology. In this study we only focus on 16S rRNA sequencing data. We have reviewed the manuscript and changed it accordingly.

*5) Availability of materials: the study is based on an open data set that is available. Availability of the source code for the experiments and results would be critical for this kind of study. I could not locate these from the manuscript. Instead, there is a text "Generated data, analysis scripts, results and supplemental information to this study will also be stored in the Fredato research data management. In case of unexpected technical limitations, we will make data, analysis scripts and supplemental information available via https://figshare.com". These materials should be made available already at the review stage, otherwise a full review is not possible.*

 The source code for this study, generated data and results will be made available at the review stage of the paper summarizing the results. At this point the source code is not complete yet, as we are only conducting the study once the protocol is accepted.

**Minor notes:**

*- Real data sets are inherently different from synthetic ones. If the underlying data distributions differ significantly between synthetic and experimental datasets, the statistical tests may yield misleading results. Some discussion could be added on this and other potential limitations of using synthetic data for methods validation.*

 According to the reviewer’s suggestion, we added a brief discussion of this aspect to the study protocol (Discussion section). A more detailed discussion will be included in the paper presenting the results of the study.

*- Possibly out of scope for the protocol study but in case all the compared data generation methods fail to produce realistic data can the authors consider suggesting their own solution to generate realistic 16S data or expand discussion on this?*

 We deliberately chose not to develop our own simulation tool in this study, as we clearly want to separate benchmarking from methods development to prevent bias. However, to maximize the realism of the synthetic data generated by the tools at hand, we already suggested to introduce two adaptations: adding zeros to the data to achieve realistic levels of sparsity and adjusting the number of differentially abundant features to better capture true effect sizes.

*- Could you consider using other similar measures to PCA, for instance, principal coordinate analysis based on different dissimilarity metrics (e.g. Jaccard and Bray-Curtis distances) to evaluate the robustness of the ordination comparison?*

 The PCA visualization we proposed will not be conducted on the count matrices, but on the characteristics calculated from those count matrices. Since it would be unusual to use the Bray-Curtis distance as a similarity measure for data characteristics and visualize it by PCoA, especially across multiple experimental datasets with differing taxonomies, we decided to incorporate the average Bray-Curtis distance between all sample pairs as an additional data property. This approach aids in assessing whether simulated count matrices are realistic.

*- Table 2: " stasticial analyses" -> "statistical analyses"?*

 Thank you, we have corrected this typo.

*Is the rationale for, and objectives of, the study clearly described?*

*Partly*

 We acknowledge that the rationale and objectives of the study may not have been communicated clearly. To clarify this, we changed the abstract. To summarize, this study is essentially a validation study, focusing on evaluating the feasibility of using synthetic data to resemble experimental data and whether we replicate findings from the reference study based multiple simulated data sets. Additionally, it is a showcase how to incorporate study protocols in computational studies and to rigorously plan a benchmark study before actually conducting it. This study serves as groundwork for a subsequent benchmark study, which will include a broader range of data sets for performance testing.

*Is the study design appropriate for the research question?*

*Partly*

 We agree that simulated data has limitations in fully validating previous findings due to inherent differences from experimental data. To address this, we calculate a comprehensive set of data characteristics, perform equivalence tests, and select only simulated data that does not show significant disagreement with experimental data. In our view, this approach is the best we can do to minimize the differences and ensure comparability between simulated and experimental datasets. Moreover, applying the presented equivalence testing procedure represents a novel methodological improvement compared to existing approaches.

*Are sufficient details of the methods provided to allow replication by others?*

*Partly*

 In the revised version we better explain especially that data generation mechanism, by including more details for the simulation process. All data sets, experimental and generated, as well as all source code to run the study will be made available once the paper describing the results will undergo review process.

*Are the datasets clearly presented in a useable and accessible format?*

*Yes*
